# Atomic-Resolution Simulations Predict a Transition State for Vesicle Fusion Defined by Contact of a Few Lipid Tails

**DOI:** 10.1371/journal.pcbi.1000829

**Published:** 2010-06-24

**Authors:** Peter M. Kasson, Erik Lindahl, Vijay S. Pande

**Affiliations:** 1 Department of Chemistry, Stanford University, Stanford, California, United States of America; 2 Center for Biomembrane Research, Stockholm University, Stockholm, Sweden; University of California San Francisco, United States of America

## Abstract

Membrane fusion is essential to both cellular vesicle trafficking and infection by enveloped viruses. While the fusion protein assemblies that catalyze fusion are readily identifiable, the specific activities of the proteins involved and nature of the membrane changes they induce remain unknown. Here, we use many atomic-resolution simulations of vesicle fusion to examine the molecular mechanisms for fusion in detail. We employ committor analysis for these million-atom vesicle fusion simulations to identify a transition state for fusion stalk formation. In our simulations, this transition state occurs when the bulk properties of each lipid bilayer remain in a lamellar state but a few hydrophobic tails bulge into the hydrophilic interface layer and make contact to nucleate a stalk. Additional simulations of influenza fusion peptides in lipid bilayers show that the peptides promote similar local protrusion of lipid tails. Comparing these two sets of simulations, we obtain a common set of structural changes between the transition state for stalk formation and the local environment of peptides known to catalyze fusion. Our results thus suggest that the specific molecular properties of individual lipids are highly important to vesicle fusion and yield an explicit structural model that could help explain the mechanism of catalysis by fusion proteins.

## Author Summary

Membrane fusion is a common underlying process critical to neurotransmitter release, cellular trafficking, and infection by many viruses. Proteins have been identified that catalyze fusion, and mutations to these proteins have yielded important information on how fusion occurs. However, the precise mechanism by which membrane fusion begins is the subject of active investigation. We have used atomic-resolution simulations to model the process of vesicle fusion and to identify a transition state for the formation of an initial fusion stalk. Doing so required substantial technical advances in combining high-performance simulation and distributed computing to analyze the transition state of a complex reaction in a large system. The transition state we identify in our simulations involves specific structural changes by a few lipid molecules. We also simulate fusion peptides from influenza hemagglutinin and show that they promote the same structural changes as are required for fusion in our model. We therefore hypothesize that these changes to individual lipid molecules may explain a portion of the catalytic activity of fusion proteins such as influenza hemagglutinin.

## Introduction

Membrane fusion is critical to eukaryotic cell function; cells rely on fusion for vesicle trafficking and secretion, and viruses such as influenza and HIV utilize fusion to infect target cells. This poses a fundamental biophysical question: how do two lipid bilayers merge in a targeted manner without rupture, and how do proteins catalyze this process? Viruses in particular are faced with a host membrane not designed to be permissive to viral entry and must alter host membrane properties to achieve fusion. Simply bringing the viral and cellular membranes together is not sufficient for physiological fusion; mutagenesis experiments in influenza [1], [2] and parainfluenza virus [3] have demonstrated that mutations to either the viral transmembrane anchor or the fusion peptide inserted in the host membrane can block fusion. In some cases [3], these mutations can be rescued by independently altering membrane properties, suggesting a direct connection between fusion peptides and lipid dynamics.

The stalk model for membrane fusion proposes that proteins catalyze the formation of a series of lipidic fusion intermediates: the outer leaflets of each bilayer merge first, followed by opening of a fusion pore and merger of the inner leaflets [4]. There is strong indirect support for this model [4]–[8], and stalk structures have been observed in artificial model systems [9], but direct observation of fusion stalks in physiological membranes is extremely challenging due to their transient nature and small size. Molecular simulations provide an alternative way to study these processes and can also provide atomic detail of the fusion mechanism and transition state, yielding insight into the mechanism of biological catalysis of fusion.

Vesicle fusion has previously been modeled with continuum approaches [8], [10]–[15] or coarse-grained simulation [16]–[19], both of which have made important contributions to refining the stalk hypothesis and outlining fusion mechanisms. One previous high-resolution simulation started from a pre-constructed stalk state, due to computational limitations, and examined a vesicle fusing to itself through a simulation boundary [20]. However, complete simulation of fusion in atomic detail has long been an important goal towards understanding atomic-level effects such as membrane dehydration and bilayer breakup upon stalk formation [21], [22].

In cells, vesicle fusion is typically catalyzed by proteins. To understand the mechanism of this catalysis, we first wish to consider the biophysical nature of fusion, its transition state, and the surrounding molecular events. We have therefore performed atomic-resolution simulations both of complete vesicles fusing and of hemagglutinin fusion peptides interacting with lipid bilayers in order to examine the mechanism of vesicle fusion and especially stalk formation in more detail. The pathway for fusion that we observe in our simulations transits through stalk and hemifused intermediates largely as predicted by the stalk hypothesis, but we observe new high-resolution details important to understanding the transition state for stalk formation and thus how fusion proteins may catalyze the fusion process.

To identify this transition state from simulations, we employ committor analysis [23]–[25], a statistical means to evaluate the transition state (as well as the full reaction pathway) that has been frequently used in the protein folding literature [23], [26], [27]. To the best of our knowledge, this marks the first time such techniques have been applied to systems of this size and complexity, simulating the million-atom vesicle fusion reaction many times over. The transition state we identify is characterized by a hydrophobic nucleation event where lipid tails from opposite vesicles make contact within the intervening hydrophilic layer. This raises the pivotal question of how fusion proteins might accelerate this hydrophobic encounter. From additional simulations of influenza fusion peptides in bilayers, we believe that fusion catalysis may be partially explained by an increased rate of hydrophobic tail protrusion in the presence of fusion peptides.

## Results/Discussion

We have simulated the fusion of vesicles using model membranes composed of binary mixtures of 1-palmitoyl 2-oleoyl phosphatidylcholine (POPC) and 1-palmitoyl 2-oleoyl phosphatidylethanolamine (POPE). These were chosen because they are the two most common non-sterol phospholipids in viral and eukaryotic cell membranes [28]–[31] and form the basis for a number of experimental fusion models, often in combination with cholesterol and sphingomyelin [32], [33]. Recent results suggest that synaptotagmin may induce membrane curvature on the order of 17 nm [34], [35]; we use 15-nm vesicles both for reasons of computational tractability and to approximate that proposed curvature. This corresponds to the small end of the size range for experimentally producible vesicles[36]. Vesicle pairs were placed at 1 nm separation and connected by a single chemical crosslinker per vesicle pair to approximate the appositional effect of fusion proteins. The total system size including solvent was just over a million atoms.

**Figure 1:**
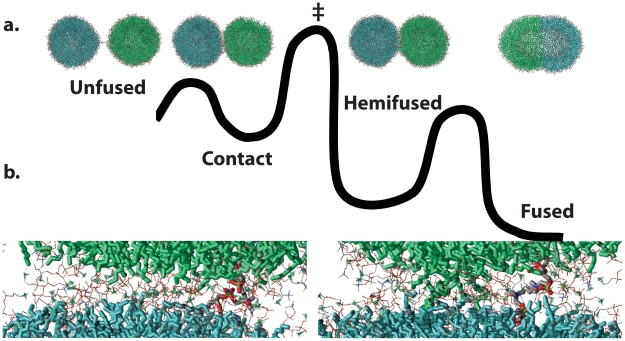
Formation of an extended contact patch between vesicles precedes fusion. Shown in (a) is a free-energy schematic for vesicle fusion, illustrated with snapshots from a fusion simulation at atomic resolution. In addition to the canonical intermediates, we find a metastable contact patch between vesicles to precede fusion. This patch is shown in (b) before (left) and after (right) stalk formation. Lipid tails are rendered in green and teal, head groups in line form. Water molecules are omitted for clarity.

Seven vesicle pairs ranging from 75–100% mole fraction POPE fused in 70–250 ns of simulation each (Figure 1); two pairs at 50% POPE did not fuse in 200 and 700 ns respectively, although we expect them to fuse in longer simulations. Aggregate simulation time totaled 10 microseconds. In all simulations, fusion occurred via initial formation of a small stalk, expansion of the hemifusion diaphragm, and subsequent opening of a fusion pore, consistent with the stalk hypothesis. In successful fusion events, vesicle pairs formed a flattened headgroup-headgroup interface with a thinned water layer prior to fusion stalk formation (Figure 1); this flattened interface greatly increases the contact area between the two vesicles, at the cost of membrane deformation. The 1∶1 POPE:POPC vesicles did not form such an interface, consistent with higher deformation energy and slower stalk formation in POPC-enriched membranes. This flattening is consistent by previous simulations by Stevens et al. [17] but was not shown in other previous coarse-grained simulations [16], [18], [37], and it has not been predicted by continuum models [11], [15]. In addition to a more detailed lipid model, our simulations use an atomic-resolution explicit solvent (the TIP3P water model [38]); desolvation effects may be important to formation of the flattened contact patch.

### Analysis of the transition state

**Figure 2:**
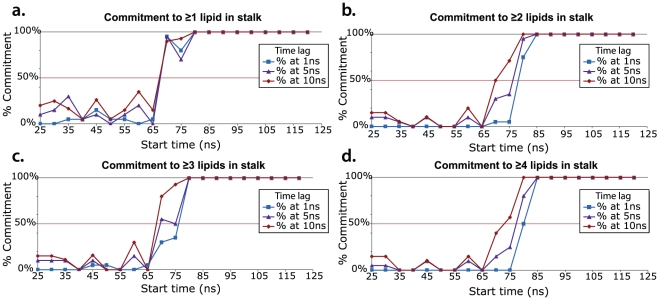
Committor analysis for transition state identification. Plots show percent commitment to the stalk state at varying time lags and thresholds for defining a stalk. To calculate these commitment probabilities, 20 snapshots were taken from a single fusion trajectory, and 20 independent simulations were started from each of these snapshots. Committor theory states that any snapshot with a 50% probability of proceeding to stalk formation is a member of the transition state ensemble [23]. To ensure robust measurement, commitment probabilities are plotted here as a function of both the length in each of these independent simulations after which stalk formation was assessed and the cutoff used to define stalk formation. Stalk formation was measured by taking 1-Å slices perpendicular to the vesicle:vesicle center-of-mass axis and measuring the number of lipids in the most sparsely populated slice, which corresponded to the polar interface region between vesicles. A stalk was judged to have formed if at least 1 lipid (**a**), 2 lipids (**b**), 3 lipids (**c**), or 4 lipids (**d**) were present in this minimal slice. The equi-commitment point or transition state occurred at approximately 75 ns (range: 65–80 ns) for all cutoffs and time lags tested.

**Figure 3:**
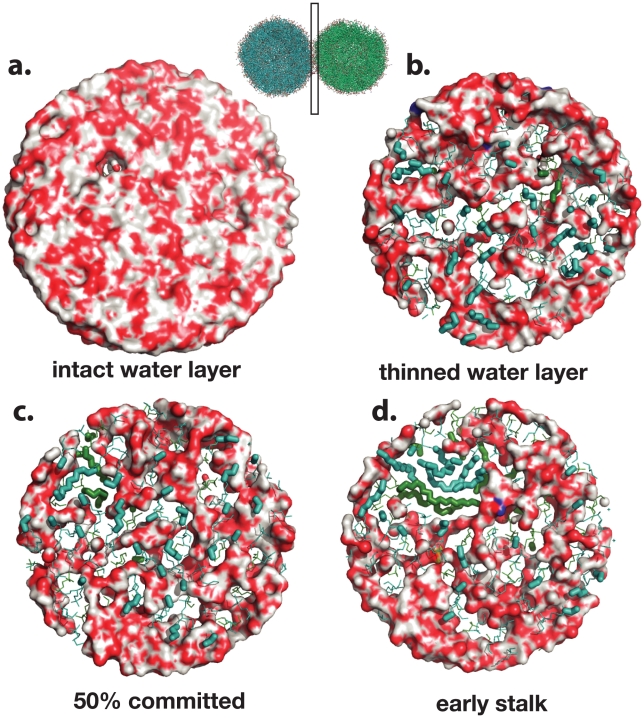
Contact patch formation is accompanied by thinning of the water layer between vesicles. Panels (a–d) show slices through the vesicle-vesicle interface at intervals around the transition state. As a contact patch forms, the water layer thins to allow contact between lipid polar headgroups. The transition state, however, does not occur until hydrophobic tails make contact. Lipids from opposing vesicles are rendered in green and teal, with head-groups rendered as lines, tails as sticks, and water in surface form. After stalk formation, the growing hydrophobic stalk excludes water from the interface region, resulting in non-leaky fusion.

**Figure 4:**
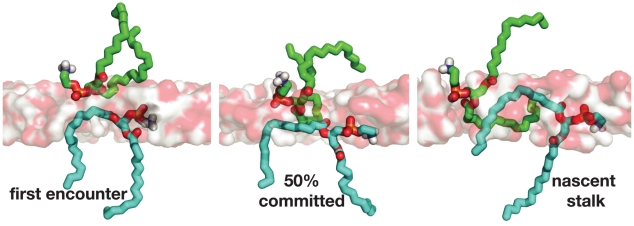
The transition state for stalk formation occurs when a pair of lipid tails make contact through the polar interface layer. In our simulations, contact between a single pair of hydrophobic tails is sufficient to nucleate stalk formation. This pair of lipids is rendered at the time of first encounter, when hydrophobic contact forms a transition state, and in the nascent stalk shortly after commitment.

**Figure 5:**
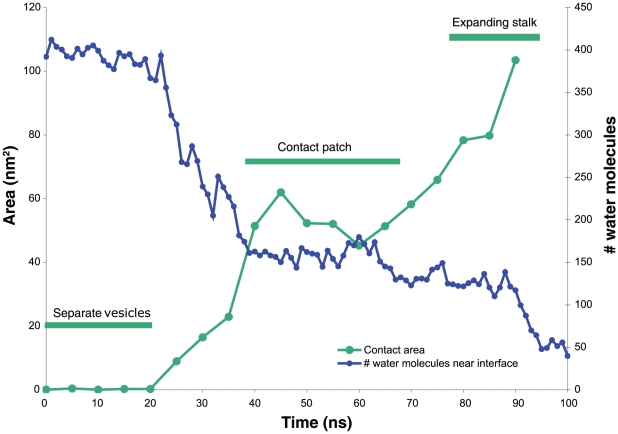
Contact area size and number of water molecules near vesicle interface. The size of the vesicle-vesicle contact area is plotted in green and the number of water molecules near the interface is plotted in blue. After the vesicles first come together, a metastable contact patch is formed where the lipid headgroups make patchy contact through a thinned water layer. The transition state for stalk formation occurs within this patch structure; later, as the stalk expands the polar contact is replaced by the nascent hemifusion structure. Contact area was measured as the difference between the solvent-accessible surface areas of the individual vesicles and the joint structure, computed with NACCESS [64]. Water molecules were counted in a cylinder of radius 2 nm and height 1 nm along the axis between the vesicle centers of mass. Lipid headgroup tilt was not measurably correlated with proximity to the vesicle-vesicle interface in our simulations (r<0.03).

**Figure 6:**
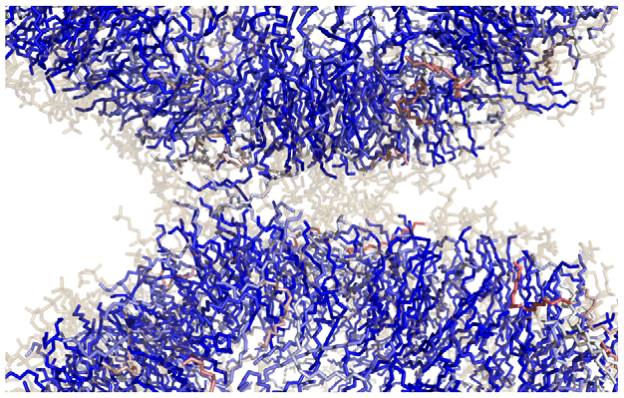
Orientation of lipid tails in a nascent fusion stalk. Most of the lipids near in this early stalk structure are either radially or tangentially aligned, not antiparallel. The stalk forms a hydrophobic “narrow bridge” between contacting vesicle outer leaflets, similar to that proposed by Kozlov and Markin[5], [12]. Outer leaflet lipids from both vesicles are shown in stick form with the lipid tails colored by the tail orientation relative to the vesicle radius. Tails aligned with the vesicle radius are colored dark blue, tails aligned antiparallel to the radius are colored dark red, and tails tangent to the vesicle are colored white. A few antiparallel tails can be seen in red, but these are generally far from the stalk. Head groups are shown in transparent brown.

To further probe the key structural features of the fusion stalk, we have performed committor analysis [23] to quantitatively identify a member of the transition state ensemble. We took simulation snapshots at 5-ns intervals from a fusion simulation and performed 20 simulations each 20 ns in length from each snapshot for a total of 400 20-ns simulations. Analysis of the resulting dataset yields the free-energy profile of a single fusion reaction (Figure 2). The transition state for stalk formation is identified via this committor analysis as the snapshot equally likely to form a stalk or remain as a contact patch. We confirm that the contact patch structure described above is metastable state or local free energy minimum, neither breaking apart rapidly nor rapidly proceeding to stalk formation. We find contact patches to be metastable for the tens to hundreds of nanoseconds, depending in part on the lipid composition. After formation of the contact patch but prior to the transition state, the water layer between vesicles thins substantially (Figure 3). The transition state occurs when a pair of lipid tails from opposing vesicles (Figure 4) make contact in the intervening polar layer. This creates a small hydrophobic region, which either breaks apart and returns to the contact patch structure or grows to form a stable stalk. Contact patch formation and water layer thinning are quantified in Figure 5. Analysis of additional independent fusion simulations confirms this lipid tail contact to be a consistent feature of stalk formation. At the time of contact, lipid tails bulge slightly into the hydrophilic layer and make contact to nucleate a stalk, but they are not grossly flipped, remaining roughly tangent to the vesicle surface (Figure 6). In our simulations, these bulging tails make contact in the polar layer between bilayers rather than inserting into the opposite bilayer. This is a difference from previous coarse-grained simulations [17] and may reflect the increased chain entropy of atomic-resolution lipids compared with coarse-grained simulations.

### A mechanism for fusion stalk formation and catalysis

These simulations suggest that the defining event for fusion occurs when two lipid tails from opposing vesicles make contact through the hydrophilic layer. To first order we can assume these bulging or protrusion events to be independent, making the nucleation probability proportional to the number of contacting lipid pairs, or equivalently the contact area A(t) at time t divided by the area per lipid head group ρ. Since this is a second-order reaction that depends on contact by two lipid tails, the nucleation probability varies with γ^2^, where γ(t) is the probability of a lipid tail bulging into the hydrophilic layer.

This model would explain why contact patch formation increases stalk formation rates; it also provides a new context to interpret the cooperative activity of fusion proteins and how they may interact with membranes. Engagement of multiple proteins, particularly in the ring arrangement proposed to drive fusion [39], will help promote larger contact patch formation, thus catalyzing stalk formation. Fusion proteins have also been proposed to catalyze fusion by disordering the lipid bilayer [40]. In our formalism, this effect could manifest as a local increase in γ, the probability of lipid tail protrusion, in the vicinity of the fusion peptides.

We use simulations to examine these hypotheses regarding lipid tail protrusion in closer detail. Both tail protrusion probability and lipid tail S_CD_ order parameters in the vesicles are uncorrelated with spatial proximity to the contact interface in our simulations (each individual correlation coefficient <0.2), so the formation of a contact patch does not itself increase protrusion rates. The uniformity of the lipid order parameters across the vesicle surface also argues against a phase change in the contact region; the contact region remains lamellar prior to stalk formation. Average SCD values from 5–15 ns prior to stalk formation are highly similar to those in planar POPC bilayers (Pearson correlation coefficient of 0.94 between values in simulated POPE vesicles and POPC bilayers measured experimentally [41]). In our simulations, therefore, fusion does not occur via a large-scale “disorientation” of lipid tails in closely apposed bilayers as has been previously suggested [42]. Lipid composition does affect protrusion, as POPE vesicles have significantly higher protrusion rates than POPC vesicles (p<0.02, Kolmogorov-Smirnov test).

**Figure 7:**
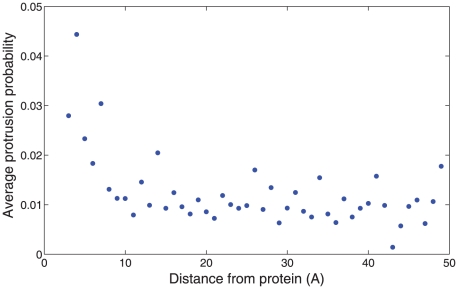
Influenza hemagglutinin peptides increase lipid tail protrusion probability. Lipid tail protrusion probability is plotted as a function of distance from the nearest fusion peptide, with values averaged in 1-Å bins. Tails within 5 Å of the fusion peptides have a significantly greater probability of protrusion than those >20 Å away (p<0.02, Kolmogorov-Smirnov test).

We also tested the ability of influenza fusion peptides to induce the tail protrusion we observe in the transition state for fusion stalk formation. Hemagglutinin fusion peptides (HA2 residues 1–20) were simulated in POPC bilayers at a peptide:lipid ratio of 3∶500 for 200 ns. Such peptides have previously been studied via molecular dynamics [43]–[46] and predicted to have a disordering effect on bilayers. In our simulations, hemagglutinin significantly increased lipid tail protrusion in nearby lipids but not the bilayer as a whole (Figure 7): lipids within 5 Å of the peptides exhibited significantly increased protrusion frequencies compared to lipids greater than 20 Å away (p<0.02 via Kolmogorov-Smirnov). No such effect was seen in membrane-inserted ion channel used as a negative control, and at >20 Å the protrusion probability was identical within error to protein-free bilayers. This local increase in protrusion does not solely account for the catalytic activity of fusion peptides, but it explains an important contribution to increased stalk formation rates. Most importantly, it provides a explicit lipid structural model for the general disordering effect that fusion peptides are thought to have on lipid bilayers to induce fusion [47].

In our simulations, fusion occurs via the following pattern: formation of a contact patch between the two vesicles precedes stalk formation. In this contact region, we observe thinning of the water layer between vesicles. Stalk formation is nucleated by a stochastic event: hydrophobic contact between a single pair of lipid tails that bulge into this water layer. If we approximate the protrusion of any single lipid tail as a statistically independent event, we derive the following model for the probability of stalk nucleation in any given time interval Δt:
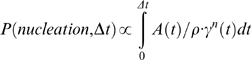
where A(*t*) is the contact patch area at time *t*, ρ is the area per lipid head group, and γ(*t*) is the probability of any single tail protruding at time *t*, and *n* is the number of contacting tails required to form the transition state. Under our simulation conditions, the transition state contains one tail from each vesicle, so in this case *n* = 2. As expected for a stochastic encounter in a planar region, most stalks form off-center. This is consistent with previous simulation reports [17] and follows trivially from our model—since P(nucleation) is proportional to the contact area A, it varies with r^2^, where r is distance to the contact patch center—but this is not how such stalks are intuitively envisioned.

The vesicles simulated here are substantially smaller than either synaptic vesicles or viral particles. This was done for reasons of computational tractability, as smaller vesicles contain fewer atoms and higher membrane curvature increases the rate of fusion pore formation. Our prediction of a flattened pre-stalk intermediate in small, highly-curved vesicles is thus particularly interesting, as flattened structures would be even more favorable in larger vesicles with lower average curvature. Compared to our small simulated vesicles, we expect physiologic fusion from a comparable activated intermediate to proceed more slowly. The kinetics of fusion are difficult to separate experimentally from the generation of an activated complex; physiologic rates have been measured as fast as ∼200 µs from calcium trigger to fusion [48] while reconstituted systems are typically slower, as fast as milliseconds for synaptic fusion [49], [50] or in the milliseconds to seconds range for viral fusion [51], [52].

These atomic-resolution simulations suggest a structural model of the transition state that could explain many aspects of fusion protein activity. To generate this structural model, we have combined parallel simulations on traditional supercomputers with many shorter simulations in a distributed computing environment to apply committor analysis to million-atom systems. In the resulting model, membrane bending by fusion protein assemblies accelerates fusion in part by driving contact patch formation. The tail protrusion induced by influenza peptides in our simulations suggests a mechanism for fusion catalysis by bilayer disordering. Other proteins such as parainfluenza virus F protein [3] and synaptotagmin [53] that are thought to catalyze fusion in part by membrane perturbation near the site of stalk formation might also act in part by catalyzing lipid tail protrusion. This suggests the hypothesis that increased lipid tail protrusion could provide a common physical mechanism of catalysis for structurally diverse proteins: class I viral fusion peptides, membrane-associated loops of class II fusion proteins, and neuronal synaptotagmin.

## Methods

Each 15-nm vesicle was composed of 877 POPC or POPE phospholipids using the Berger simulation parameters [54]. The crosslinker structure was -CO(CH2)_4_CO-, connected to POPC lipids via an amide linkage to the headgroup nitrogen. Individual vesicles were first equilibrated in the TIP3P explicit solvent model of water [38]. Pairs of vesicles were then placed at 1 nm separation in a hexagonal box with sides 21 nm and height 32.5 nm and solvated in TIP3P water with or without 150 mM NaCl, leading to a system size of over a million atoms. Simulations were run using Gromacs 4.0 [55] under constant temperature and pressure using Berendsen pressure coupling and the velocity-rescaling thermostat at 310 K [56]. All covalent bond lengths were constrained using LINCS [57], and long-range electrostatics were computed every step using Particle Mesh Ewald (PME) [58]. The amine hydrogen atoms on POPE were converted to virtual interaction sites [55] to enable longer time steps by constraining the only polar hydrogens in the lipid system. The atomic coordinates are constructed every step, and forces acting on them are interpolated back onto the mass centers. This approach has been shown to conserve energy [59], but we also checked the model by testing both 2 fs or 4 fs timesteps, with equivalent results for a pair of full fusion trajectories.

Hemagglutinin fusion peptides were simulated based on PDB structure 1IBN [60] using the AMBER03 force field to model the amino acids [61]. 3 copies of the fusion peptide were placed in a bilayer as reported previously [44] for a total peptide:lipid ratio of 3∶500, solvated with TIP3P water and 150 mM NaCl, and simulated for 200 ns using 2 fs timesteps, PME electrostatics, and constant pressure and temperature conditions with at 300 K with semi-isotropic pressure coupling. Lipid tail protrusion rates were significantly increased for lipids with an average distance of 5 Å to the closest peptide atom (p<0.02, Kolmogorov-Smirnov test). Simulations of a GLIC ion channel based on PDB structure 3EI0 [62] in a POPC bilayer were used as a negative control and showed no such increase. Tail protrusion was defined as any carbon in the lipid tail protruding more than 1 Å beyond the phosphate group.

Each long fusion simulation was run on 128 cores of a cluster using Intel Clovertown or Harpertown CPU's respectively connected by an Infiniband network; the 400 “shooting” simulations were each run on 8–16 cores using the Folding@Home distributed computing network [63]. The aggregate length of vesicle fusion simulations was 10 microseconds.
